# Investigation of allosteric modulation mechanism of metabotropic glutamate receptor 1 by molecular dynamics simulations, free energy and weak interaction analysis

**DOI:** 10.1038/srep21763

**Published:** 2016-02-18

**Authors:** Qifeng Bai, Xiaojun Yao

**Affiliations:** 1 Key Laboratory of Preclinical Study for New Drugs of Gansu Province, School of Basic Medical Sciences, Lanzhou University, Lanzhou, Gansu 730000, P. R. China; 2 Department of Chemistry, Lanzhou University, Lanzhou 730000, China

## Abstract

Metabotropic glutamate receptor 1 (mGlu_1_), which belongs to class C G protein-coupled receptors (GPCRs), can be coupled with G protein to transfer extracellular signal by dimerization and allosteric regulation. Unraveling the dimer packing and allosteric mechanism can be of great help for understanding specific regulatory mechanism and designing more potential negative allosteric modulator (NAM). Here, we report molecular dynamics simulation studies of the modulation mechanism of FITM on the wild type, T815M and Y805A mutants of mGlu_1_ through weak interaction analysis and free energy calculation. The weak interaction analysis demonstrates that van der Waals (vdW) and hydrogen bonding play an important role on the dimer packing between six cholesterol molecules and mGlu_1_ as well as the interaction between allosteric sites T815, Y805 and FITM in wild type, T815M and Y805A mutants of mGlu_1_. Besides, the results of free energy calculations indicate that secondary binding pocket is mainly formed by the residues Thr748, Cys746, Lys811 and Ser735 except for FITM-bound pocket in crystal structure. Our results can not only reveal the dimer packing and allosteric regulation mechanism, but also can supply useful information for the design of potential NAM of mGlu_1_.

The metabotropic glutamate receptor 1 (mGlu_1_) is one member of class C G protein-coupled receptors (GPCRs) and is considered to be a promising target to treat Alzheimer’s disease, anxiety, cancer, schizophrenia autism and chronic pain^1^,^2^,^3^. It can form constitutive homodimer to activate the downstream signal modulator at the cell surface^4^. The mGlu_1_ receptor contains orthosteric binding site in Venus flytrap domain (VFD) and allosteric binding site in the transmembrane (7TM) domain^3^. The orthosteric ligands can activate or inactivate the mGlu_1_ through binding to VFD. The allosteric modulators can alter the binding affinity of orthosteric ligands in negative, neutral and positive ways by binding to allosteric sites of mGlu_1_^4^,^5^. Some recent studies prove that only a single 7TM is promoted to couple with G protein in the agonist-bound homodimeric mGlu_1_^6^,^7^,^8^.

The recent reported crystal structure of mGlu_1_ has provided useful information to understand the structural features and activation mechanism of class C GPCRs^3^,^9^. However, it is still difficult to know about the dynamical structural characteristics of mGlu_1_ at atomic level based on the crystal structure. As a complementary method to experimental study, molecular dynamics (MD) simulations can provide a useful and reliable way to study the dynamical behavior of mGlu_1_ at atomic level. Computational methods have also been successfully used to predict the thermostable mutants of mGlu_1_ to form a single stable crystal conformational state bound to ligands^10^. MD and metadynamics simulations are also proved to be very useful to understand the regulation mechanism of smoothened (SMO) receptor, which has the similar long and narrow pocket with mGlu_1_ in the 7TM domain^11^. Based on the known crystal structures and MD simulation, MD simulation methods have been successfully used to study the dynamical behavior and elucidate the allosteric regulation mechanism^12^,^13^,^14^,^15^,^16^,^17^,^18^.

The mGlu_1_ has been considered to be an important target of the known structure of class C GPCRs for structure-based drug design^19^. Due to the specific allosteric sites in the 7TM domain of mGlu_1_, negative allosteric modulators (NAM) can be designed based on the crystal structure or known NAM^3^,^20^,^21^. The cholesterols are considered as the lipid rafts to pack the lipids and membrane proteins tightly^22^. The dynamical dimer mediated by cholesterols and allosteric mechanism of mGlu_1_ are still elusive. It is difficult to distinguish the main interactive way such as hydrogen bonding, steric repulsion or van der Waals (vdW) between mGlu_1_ and NAM. It is worthwhile to study the detailed dynamical dimer interaction and allosteric mechanism between the homodimers of mGlu_1_ and allosteric ligands.

Here, we perform molecular dynamics simulations on wild type, T815M and Y805A mutants of mGlu_1_. The weak interaction and free energy calculation are employed to analyze the dimerization and allosteric mechanism. The results of weak interaction analysis indicate that T815 and Y805 in wild type mGlu_1_ have stronger vdW and hydrogen bonding interaction than that in T815M and Y805A mutants of mGlu_1_. The free energy profiles energy barrier in the first and secondary pockets for binding of FITM. Our results supply a detail structural basis for the allosteric and homodimer mechanism of mGlu_1_.

## Methods

### Model preparation

To study the dimer structure of metabotropic glutamate receptor 1 (mGlu_1_), the crystal structure of mGlu_1_ is obtained from PDB database (ID: 4OR2)^3^. The redundant residues are deleted except mGlu_1_ and its ligand. The dimer of mGlu_1_ is surrounded by the explicit 1-palmitoyl-2-oleoyl-sn-glycero-3-phosphocholine (POPC) lipids with length of 68 Å and width of 101 Å. The complex of lipid and protein is immersed into TIP3P water^23^ box with the size of 68 Å × 101 Å × 90 Å. The ionic concentration of system is set to be 0.15mol/L according to the physiological ion concentration and neutralized by the sodium and chloride ions. The final system contains a total of ~46,000 atoms per periodic cell. The charges and Lennard-Jones parameters are assigned according to the atomic types which are defined in CHARMM force field. The CHARMM force field parameterizations of (4-fluoro-N-(4-(6-(isopropylamino)pyrimidin-4-yl)thiazol-2-yl)-N-methylbenzamide) FITM are generated by using CGenFF^24^ and SwissParam^25^. Once the parameters of models are completely built, the T815 and Y805 are mutated into M815 and A805 to construct another two simulated systems by using VMD software^26^.

### Molecular dynamics simulations

To equilibrate the wild type and two mutants of mGlu_1_ in complex with FITM, firstly, for each system, all atoms are fixed except for lipid tail to minimize 10 ps and equilibrate 500 ps. Secondly, the mGlu_1_ and FITM are constrained, the system is minimized for 10 ps and equilibrated for 500 ps. Thirdly, these three entire systems are all released to carried out 5 ns equilibrated MD simulations. At last, the 50 ns MD simulations are performed on these three systems under constant temperature (310 K) and constant pressure (1 bar).

Our MD simulations are run with the time step of 2 fs by using NAMD^27^ (version 2.9b3) software with CHARMM 27 force field^28^ in periodically infinite explicit water and lipid. The conjugate gradient method is employed for the minimization. The electrostatic interaction is computed with a 12 Å nonbonded cutoff based on the particle-mesh Ewald (PME)^29^ method. All MD simulations are based on the langevin thermostat and langevin barostat^30^ methods for the constant temperature of 310 K and pressure of 1 bar, respectively. The simulated trajectory frames are saved every 5 ps for analysis.

### Weak interaction analysis

The weak interaction, which involves noncovalent interaction^31^,^32^, can be used to find the main interactive way between receptor and ligand from hydrogen bonding, electrostatic, steric repulsion and van der Waals (vdW) forces. The hydrogen bonds occupancy are popularly used to study the interaction between the ligands and receptors^33^. However, the hydrogen bonds occupancy can not distinguish other noncovalent interaction between protein and ligands such as steric repulsion and vdW forces and vdW may not contribute a favorable force on the binding between receptor and ligands. The weak interaction can detail the favorable and unfavorable contributions of protein-protein, protein-ligand or protein and nucleic acids clearly. The weak interaction analysis can be shown by plotting reduced density gradient (RDG or s(**r**)) with respect to the function of electron density ρ(r) (see eq. 1):


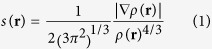


However, the weak interaction on one frame of trajectory reflects the limited information for protein complex. The average weak interaction, which bases on the full trajectory of molecular dynamics simulations, can provide the more accuracy and smooth isosurfaces between protein and protein or protein and ligand. The average weak interaction analysis^34^ is calculated based on averaged density gradient 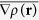 and averaged density 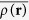 (see eq. 2):


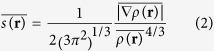


The Multiwfn^35^ software is used to analyze the weak interaction for wild type and mutated mGlu_1_ in complex with FITM. The number of grids is set to 200 × 200 × 200 in three dimensional spaces. The 5000 frames of trajectories are extracted to average the density. The representation and color of averaged weak interaction are shown in VMD software^26^. To analyze traditional hydrogen bonds occupancy, the angle and distance between the donors and acceptors are set no more than 35° and 3.5 Å, respectively^36^.

### Free energy calculation

Adaptive biasing force (ABF)^37^,^38^ are popularly used to calculate the free energy during the dissociation process of ligand in the pocket of protein. The equal probability and reversible process sampling are used to improve accuracy of free energy calculation along reaction coordinate. The free energy ΔG is obtained from the reaction coordinate a to b as shown in equation 3:


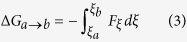


where *F*_ξ_ is the biasing force. Because the model of mGlu_1_ is constructed along Z axis, the free energy of FITM is calculated along Z axis in this study. To study the dissociation process of FITM in the pocket of mGlu_1_, the reference residues are chosen Cys671, Thr793, Thr824 and Leu763. The reaction coordinate of ABF is divided into three non-overlapping windows. Each window is set to 8 Å with 0.2 Å/bin. The wall force constant is assigned to 50 kcal/mol/Å^2^ for the boundary potential. The 500 samples are applied before a biasing force. The 20 ns MD simulations are carried out on each window. A total of 360 ns ABF simulations are performed on the systems of wild type and mutated mGlu_1_ in complex with FITM.

## Results and Discussion

### Structural features and fluctuation of mGlu_1_ dimers

**Figure 1:**
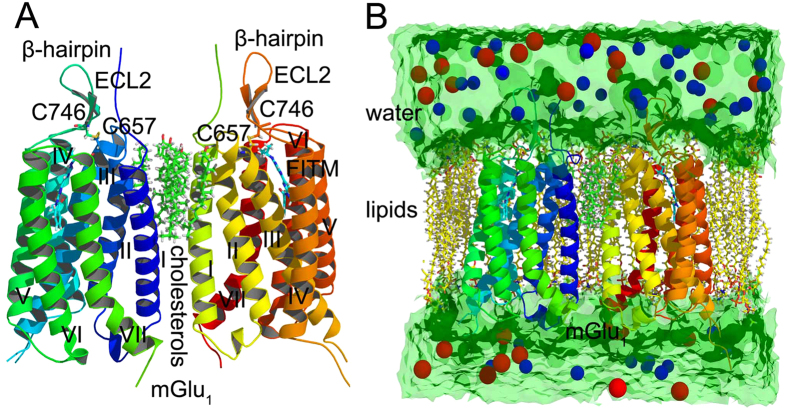
The dimer structure of mGlu_1_. (**A**) The cartoon representation of mGlu_1_. (**B**) The model of mGlu_1_ in complex with ions, FITM, cholesterols, lipids and water. The red and blue spheres are the sodium and chloride ions.

The function structure of mGlu_1_ is formed by asymmetric parallel monomer which contacts with each other by six cholesterol molecules packed against the helices I and II of mGlu_1_ (Fig. 1A,B). In the extracellular part of mGlu_1_, residue C657 forms disulfide bond with C746 to connect the β-hairpin and helix III. The negative allosteric modulator FITM locates at the pocket which surrounds by seven helixes of mGlu_1_ (Fig. 1A).

**Figure 2:**
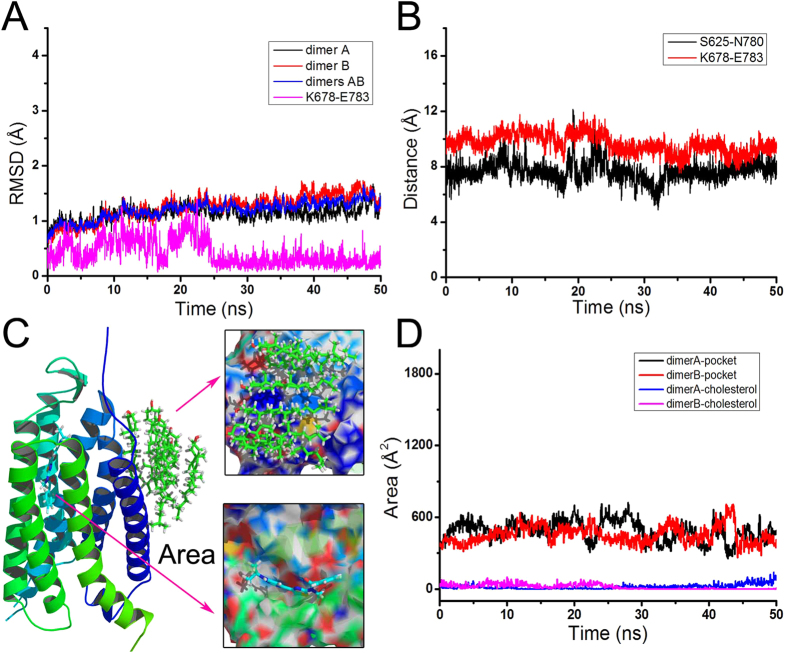
The structural fluctuation of mGlu_1_. (**A**) The RMSD of backbone atoms of dimer A, B, residues K678-E783 and two dimers of wild type mGlu_1_ versus simulation time. (**B**) The distances of S625-N780 and K678-E783. (**C**) The cartoon representation of occupied surface area of FITM and six cholesterols on mGlu_1_. (**D**) The occupied surface area of FITM and six cholesterols with respect to simulation time in dimer A and B, respectively.

To study the structural fluctuations of the entire system, the wild type, T815M and Y805A mutants of mGlu_1_ in complex with ions, FITM, cholesterols, lipids and water are built as the initial structure for molecular dynamics simulations (Fig. 1B). As shown in Fig. 2A, the root mean square deviation (RMSD) of backbone atoms of dimer A, B or the entire mGlu_1_ (dimer A and B) indicates that the whole system reaches equilibrium phase in the 50 ns simulation time. The residues K678 and E783 are considered as “ionic lock” between helix III and VI of mGlu_1_. The RMSD and distance of K678-E783 indicate the mGlu_1_ still keep stable inactive conformation during the molecular dynamics simulations (see Fig. 2A,B). Besides, the residues S625 and N780 have interaction in the intercellular part of mGlu_1_. Figure 2B shows S625 keeps stable distance with N780 in 50 ns molecular dynamics simulations. These results indicate that the mGlu_1_ systems have reached the equilibrium phase during the 50 ns molecular dynamics simulations.

### The weak interaction analysis for cholesterols in wild type mGlu_1_

The mGlu_1_ is in form of dimer A and B which interact with each other by six cholesterol molecules. The ligand FITM is a negative allosteric modulator (NAM) which can alter the activity of mGlu_1_ through interaction with the allosteric residues. The cholesterols and FITM studies can profile the dimerization and allosteric regulation mechanism of mGlu_1_. As shown in Fig. 2C,D, the occupied surface areas of ligand FITM and six cholesterol molecules on mGlu_1_ are calculated by VolArea^39^. The cholesterol molecules occupy an area about 450 Å^2^ in dimer A or B during the molecular dynamics simulations. It indicates that the cholesterol molecules can interact with dimer A and B. The allosteric modulator FITM also possesses relatively stable area in the pocket of mGlu_1_. It indicates that cholesterol molecules and FITM can form the dynamical stable interaction with mGlu_1_.

**Figure 3:**
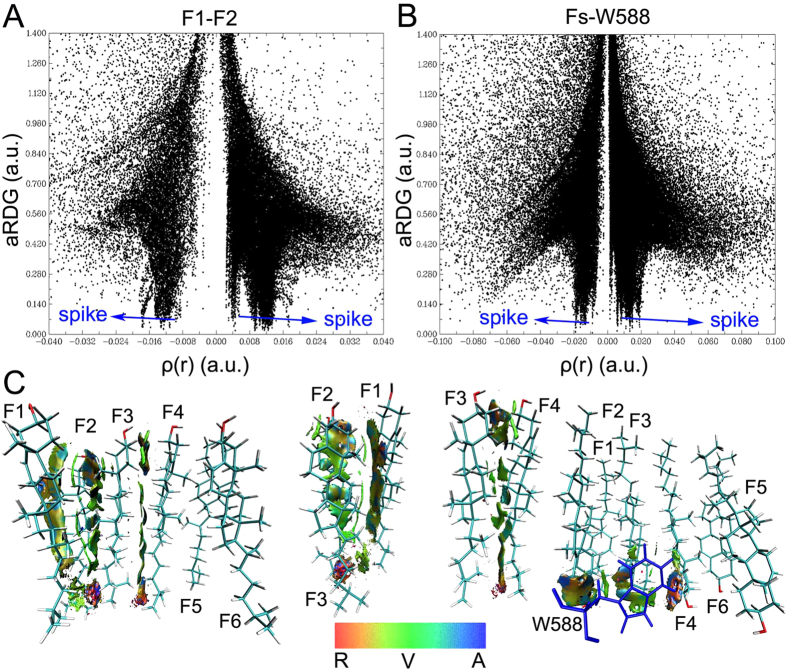
The weak interaction analysis for cholesterol fragments. (**A**) The average reduced density gradient (aRDG) versus average effective density between cholesterol fragment 1 (F1) and 2 (F2). (**B**) The average reduced density gradient (aRDG) versus average effective density between fragments 1, 2, 3, 4, 5, 6 (Fs) and residue W588. (**C**) Gradient isosurfaces for F1, F2, F3, F4 and residue W588. The color bar shows the blue-green-red scale ranging from −0.04 to 0.02 au. Blue indicates the strong attractive interaction, while red indicates repulsive interaction. The letters A, R and V in color bar represents attraction, repulsion and van der Waals interaction.

The weak interaction analysis can detail favorable and unfavorable interactions for protein and ligand. It can also complements with hydrogen bonding analysis, steric repulsion and van der Waals (vdW) interaction. Here, we employ the weak interaction analysis to profile the dimers packing and mGlu_1_-cholesterols interaction mechanism. Due to the plane symmetry on six cholesterol fragments (Fs), the fragments 1-4 (F1-4) are chosen to analyze the weak interaction. The average reduced density gradient (aRDG) of F1-4, Fs and W588 is calculated versus averaging effective density based on molecular dynamics simulations (Fig. 3A,B and Supplementary Figure S1). The left and right spikes indicate the attracting and steric repulsive effects between two molecules, respectively. As shown in Fig. 3C, the green and light blue are major colors on isosurfaces between the major parts of cholesterol fragments. It indicates that the vdW and weak hydrogen bonds play an important role on the packing of main part of cholesterol fragments. The part of head and tail of cholesterol fragments shows the red and orange. It indicates the head and tail parts have the dynamical friction between cholesterol fragments during the molecular dynamics simulations. W588 is considered as an important residue to interact with six cholesterol molecules^3^. Figure 3C shows W588 in dimer A have strong hydrogen bonding and vdW interaction with F1-F3. The isosurfaces show the steric repulsive effect between F4 and W588. However, according to the symmetry, the W588 in dimer B can interact with F4-F6 through hydrogen bond and vdW interaction. Two residues W588 of dimer A and B are like the buttons to connect with each other tightly by cholesterol molecules. The results indicate that the vdW and hydrogen bonding can pack the main parts of six cholesterol molecules and mGlu_1_ stably.

### The weak interaction analysis for FITM in wild type and mutated mGlu_1_

**Figure 4:**
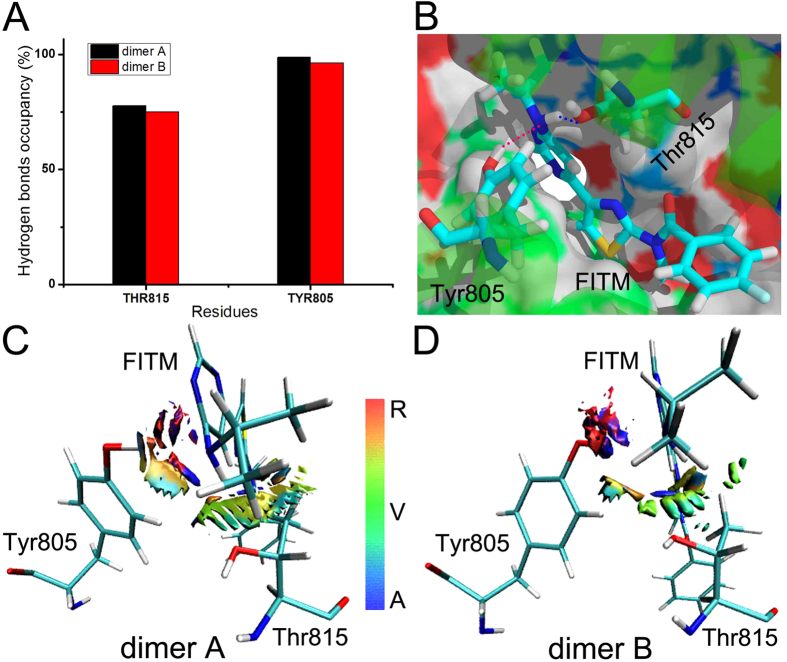
The weak interaction analysis in wild type mGlu_1_. (**A**) The hydrogen bonds occupancy between key residues Thr815, Tyr805 of dimer A, B and NAM FITM in 50 ns simulation time. (**B**) The representation of hydrogen bonds between residues Thr815, Tyr805 and NAM FITM. (**C,D**) Gradient isosurfaces between residues Thr815, Tyr805 and NAM FITM in dimer A and B of mGlu_1_.

The residues Thr815 and Tyr805 are the notable and key sites for the allosteric modulation of FITM in mGlu_1_^3^. To the study the allosteric modulation mechanism of mGlu_1_, the weak interaction analysis is employed. As shown in Fig. 4A,B, FITM has the high hydrogen bonds occupancy with Thr815 and Tyr805 in dimer A and B of mGlu_1_ during molecular dynamics simulations. The nitrogen and hydrogen atoms of FITM form the dynamical hydrogen bonds with the hydrogen atom of Tyr805 and oxygen atom of Thr815, respectively (see Fig. 4B). It indicates that there is a strong attraction interaction between FITM and allosteric sites. To explore other possible interaction mode between FITM and residues Thr815, Tyr805, the weak interaction analysis is used to generate the gradient isosurfaces between residues Thr815, Tyr805 and allosteric modulator FITM in dimer A and B. The scatter diagram is drawn to show the spikes which represent the weak interaction between FITM and residues of dimer A and B (Supplementary Figure S2). It is obviously that the vdW effect is also the main interactive way between FITM and allosteric residues Thr815 and Tyr805 (see Fig. 4C,D). Some parts of gradient isosurfaces show orange and red between FITM and allosteric residues. It indicates that there is the steric repulsion between FITM and allosteric residues during the molecular dynamics simulations. However, the attracted force is greater than the repulsive force totally. These results show that vdW and hydrogen bonds effects can keep the stable allosteric binding between FITM and residues Thr815, Tyr805 together even though some repulsive interaction exists between FITM and allosteric sites during molecular dynamics simulations.

**Figure 5:**
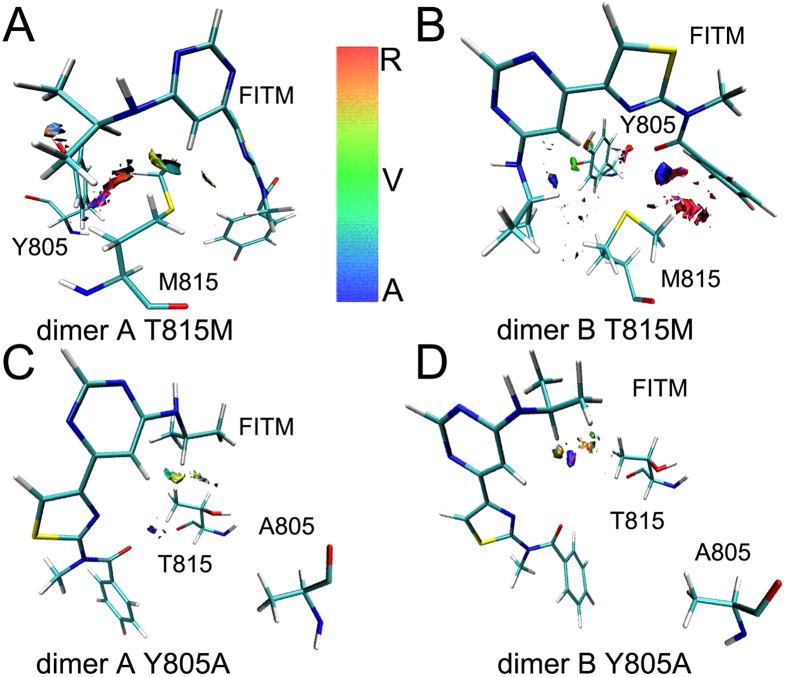
The weak interaction analysis in T815M and Y805A mutants of mGlu_1_. (**A,B**) Gradient isosurfaces between residues Met815, Tyr805 and FITM of dimer A, B in T815M mutant of mGlu_1_. (**C,D**) Gradient isosurfaces between residues Thr815, Ala805 and FITM of dimer A, B in Y805A mutant of mGlu_1_.

The T815M and Y805A mutants are reported to notably reduce the binding affinity of FITM^3^. To further detail allosteric mechanism of FITM in mGlu_1_, 100 ns MD simulations are further performed on the mutated systems. The RMSDs of T815M and Y805A mutants show the mutated systems get into the equilibrium phase during 50 ns MD simulations (Supplementary Figure S3). The weak interaction analysis is calculated based on the simulation trajectories (Fig. 5A–D and Supplementary Figure S4). In the T815M mutant, the residue M815 of mGlu_1_ forms two different conformations to interact with FITM in dimer A and B. As shown in Fig. 5A, because the methyl group of M815 turns round to FITM in dimer A, M815 has the weak vdW and hydrogen bonds with FITM except the strong repulsive interaction. In dimer B, the methyl group of M815 stays away from FITM and it does not show the vdW effect except attracting and strong repulsive interaction between M815 and FTIM (Fig. 5B). The small blue and green gradient isosurfaces indicate that the vdW and hydrogen bonds forces are also reduced between allosteric residue Y805 and FITM in the T815M mutant during molecular dynamics simulations. It infers that the M815 has longer side chain than T815 to enhance the steric repulsion between FITM and allosteric sites.

**Figure 6:**
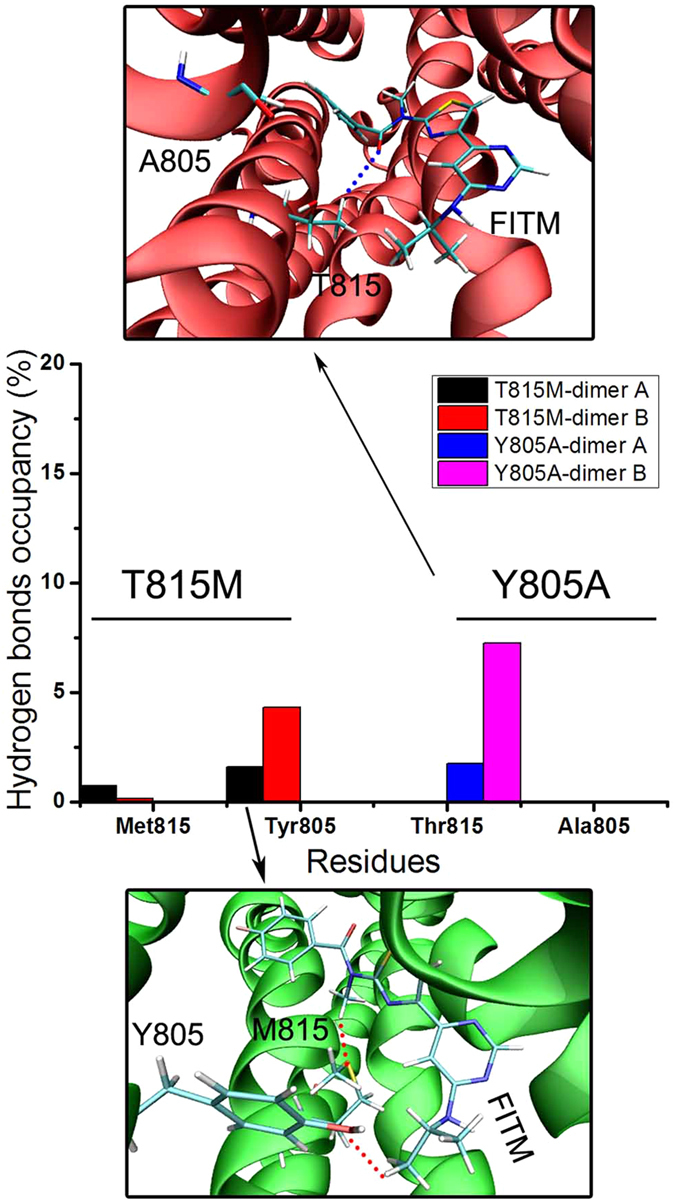
The hydrogen bonds occupancy between FITM and key residues T815, M815, Y805, A805 during molecular dynamics simulations.

In the Y805A mutant, the residue A805 has no hydrogen bond and vdW interaction with FITM in mGlu_1_ during molecular dynamics simulations (see Fig. 5C,D). The green and blue, which only occupancy few area between residue T815 and FITM, indicate that T815 in the Y805A mutant has very weak vdW and hydrogen bonds interaction with FITM compared with T815 in wild type mGlu_1_ (Figs 4C,D and 5C,D). The hydrogen bonds occupancy is used to analyze the interaction between residues M815, A805 and FITM. As shown in Fig. 6, in T815M mutant, the residues M815 occupy less than 1% hydrogen bonds occupancy in dimer A and B during molecular dynamics simulations. In Y805A mutant, the residue A805 does not form the hydrogen bonds with FITM with molecular dynamics simulations. The residue T815 forms less than 9% hydrogen bonds occupancy during molecular dynamics simulations. The results of weak interaction analysis and hydrogen bonds occupancy analysis show that the allosteric sites T815 and Y805 are important residues for negative allosteric modulator design of mGlu_1_.

### Free energy calculations

**Figure 7:**
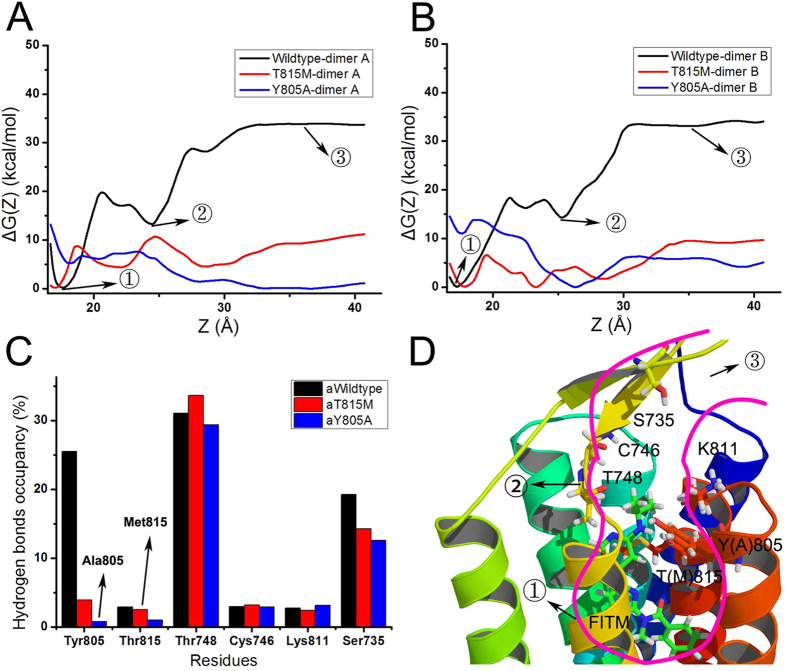
Free energy calculations. (**A,B**) The free energy calculations are profiled when FITM separates from dimer A, B of wild type, T815M and Y805A mGlu_1_ system, respectively. (**C**) The average hydrogen bonds occupancy on dimer A and B of mGlu_1_ along the separated route of FITM. (**D**) The cartoon diagram of the separated path of FITM and key interactive residues of overlapped structures of wild type, T815M and Y805A mutants of mGlu_1_. The T(M)815 represents residue T815 or M815. And Y(A)805 represents residue Y805 or A805.

The weak interaction analysis can provide information about the interaction between allosteric ligand and key residues in the binding pocket of mGlu_1_. Free energy calculation can enhance the understanding of the NAM behavior during the dissociation process of FITM in the binding pocket of mGlu_1_. Adaptive biasing force (ABF) is employed here to calculate the free energy when FITM dissociates from dimer A and B of mGlu_1_. Figure 7A,B shows the free energy of FITM during the dissociation process from the pocket of wild type, T815M and Y805A mutants of mGlu_1_, respectively. It need conquer about 35 kcal/mol energy when FITM gets out of the pocket in dimer A and B of wild type mGlu_1_. It is obviously that FITM in dimer A and B of wild type mGlu_1_ need overcome higher barrier energy than in dimer A and B of T815M and Y805A mutants of mGlu_1_. It indicates that FITM tends to bind the pocket of wild type mGlu_1_ than in T815M and Y805A mutants of mGlu_1_. The average hydrogen bonding occupancies of wild type, T815M and Y805A (aWildtype, aT815M, aY805A) mutants of mGlu_1_ are calculated between FITM and mGlu_1_ along the dissociation route of FITM (Fig. 7C,D). The dynamical dissociation process of FITM from the pocket of mGlu_1_ is shown in Supplementary Movie S1. The main residues, which interact with FITM along the dissociation path, are Tyr805, Thr815, Thr748, Cys746, Lys811 and Ser735. It is obviously that the residue Tyr805 in wild type of mGlu_1_ has higher hydrogen bond occupancy than that in T815M and Y805A mutants of mGlu_1_ (Fig. 7C). Due to the small volume of Ala805, FITM forms the lowest number of hydrogen bonds with Ala805 in Y805A mutant. Residue Thr748 in the extracellular part has the higher hydrogen bonds occupancy in wild type, T815M and Y805A mutants of mGlu_1_ because the residue Thr748 forms a secondary pocket with Lys811, Cys746 and Ser735 in β-hairpin above the pocket of mGlu_1_ (Fig. 7C,D).

Figure 7A,B and D show the two main energy deep wells in dimer A and B of wild type mGlu_1_. The lowest energy deep well corresponds to the FITM-bound pocket in the crystal structure of mGlu_1_ in the first state. FITM binds to allosteric site residues T815 and Y805 stably. The state 2 shows that FITM places into another higher energy deep well after conquering the energy barrier of ~20 kcal/mol. FITM binds to a secondary pocket which mainly forms by the residues Thr748, Cys746, Lys811 and Ser735. In the state 3, the FITM has been pulled out of mGlu_1_ completely through crossing over ~20 kcal/mol energy barrier. It can be seen that the residues in secondary pocket play an important role on the dynamical binding between mGlu_1_ and negative allosteric modulator.

## Conclusions

In this work, we studied the dimeric packing and allosteric regulation mechanism of mGlu_1_ through weak interaction analysis and free energy calculation based on the molecular dynamics simulations. The results from MD simulation and weak interaction analysis indicate that vdW and hydrogen bond interaction play an important role on the packing between six cholesterol molecules and the dimer of mGlu_1_. Besides, the weak interaction analysis also indicates that residues T815 and Y805 in wild type mGlu_1_ can form stronger vdW and hydrogen bonding interaction with FITM than that in T815M and Y805A mutants of mGlu_1_. The free energy calculation shows that residues Thr748, Cys746, Lys811 and Ser735 are important residues before the ligand escapes from the pocket of mGlu_1_. Our results reveal the dimer packing and allosteric mechanism of mGlu_1_, which will be useful for the allosteric modulator design.

## Additional Information

**How to cite this article**: Bai, Q. and Yao, X. Investigation of allosteric modulation mechanism of metabotropic glutamate receptor 1 by molecular dynamics simulations, free energy and weak interaction analysis. *Sci. Rep.* **6**, 21763; doi: 10.1038/srep21763 (2016).

## Supplementary Material

Supplementary Information

Supplementary Movie S1
