# Supplementary material for: Investigation of allosteric modulation mechanism of metabotropic glutamate receptor 1 by molecular dynamics simulations, free energy and weak interaction analysis
Source: Sci Rep. 2016 Feb 18;6:21763. doi: 10.1038/srep21763 (PMC4757871; doi:10.1038/srep21763)
Supplement: Supplementary Information [file srep21763-s1.doc]

**Investigation of allosteric modulation mechanism of metabotropic glutamate receptor 1 by molecular dynamics simulations, free energy and weak interaction analysis**

Qifeng Bai^1^, Xiaojun Yao^1,2*^

^1^Key Laboratory of Preclinical Study for New Drugs of Gansu Province, School of Basic Medical Sciences, Lanzhou University

^2^Department of Chemistry, Lanzhou University, Lanzhou 730000, China


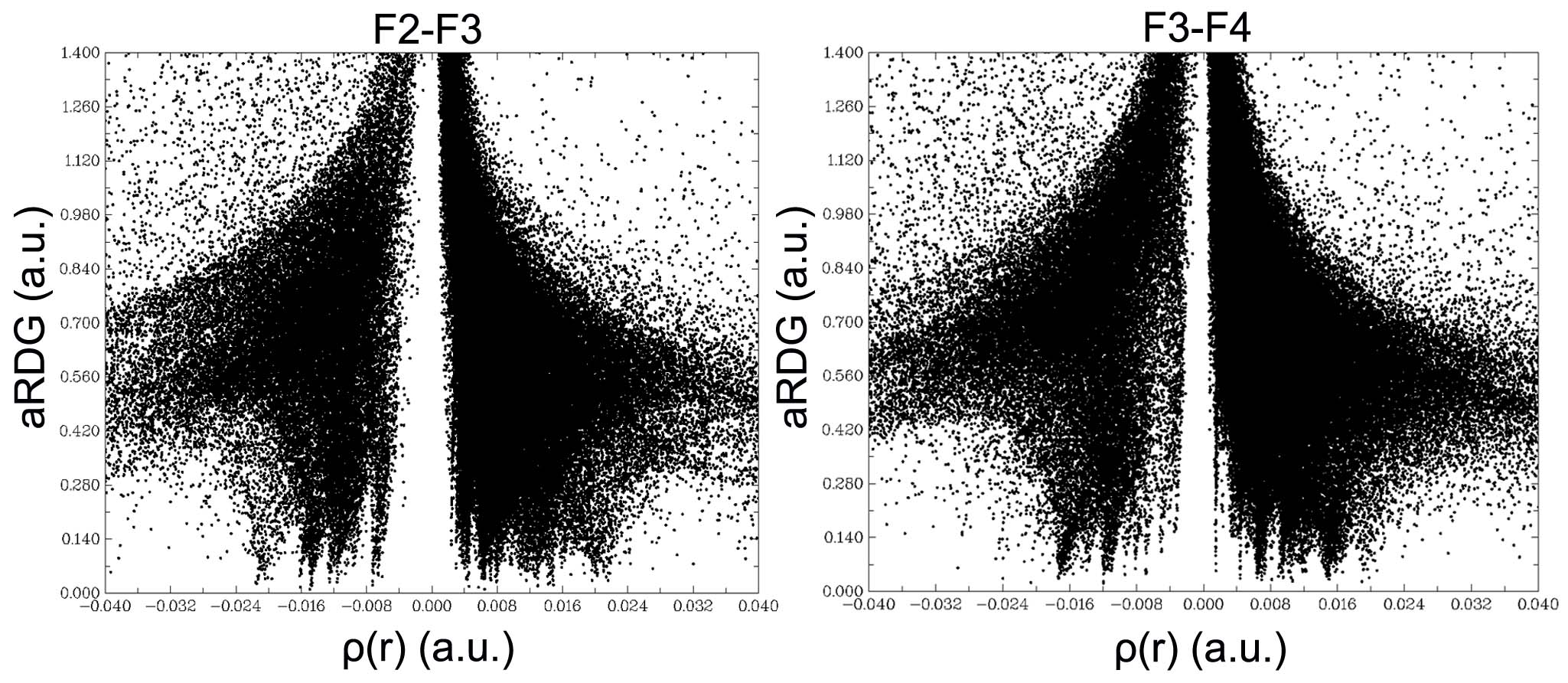


**Figure S1.** The averaging reduced density gradient (aRDG) versus averaging effective density between cholesterol fragment F2-F3 and F3-F4.


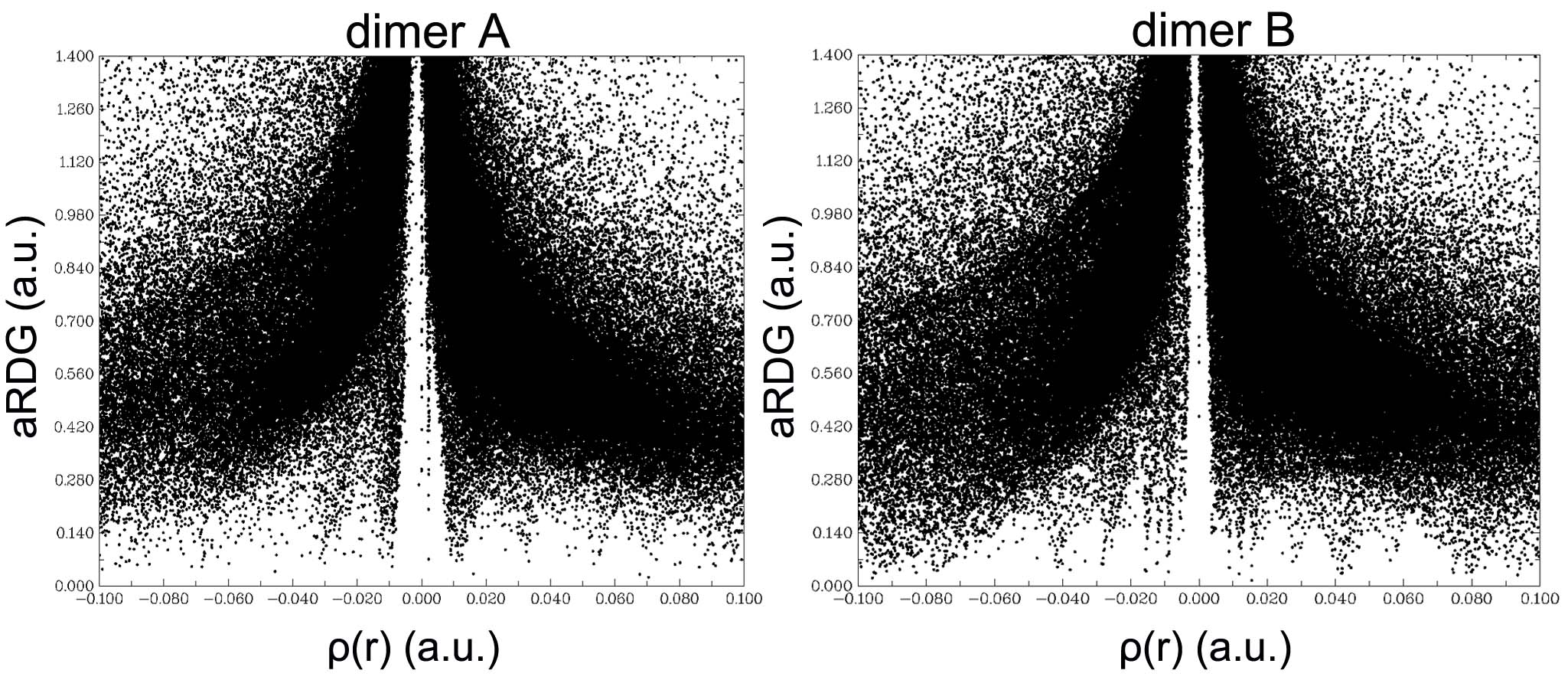


**Figure S2.** The averaging reduced density gradient (aRDG) versus averaging effective density between FITM and dimer A, B in wild type mGlu_1_, respectively.


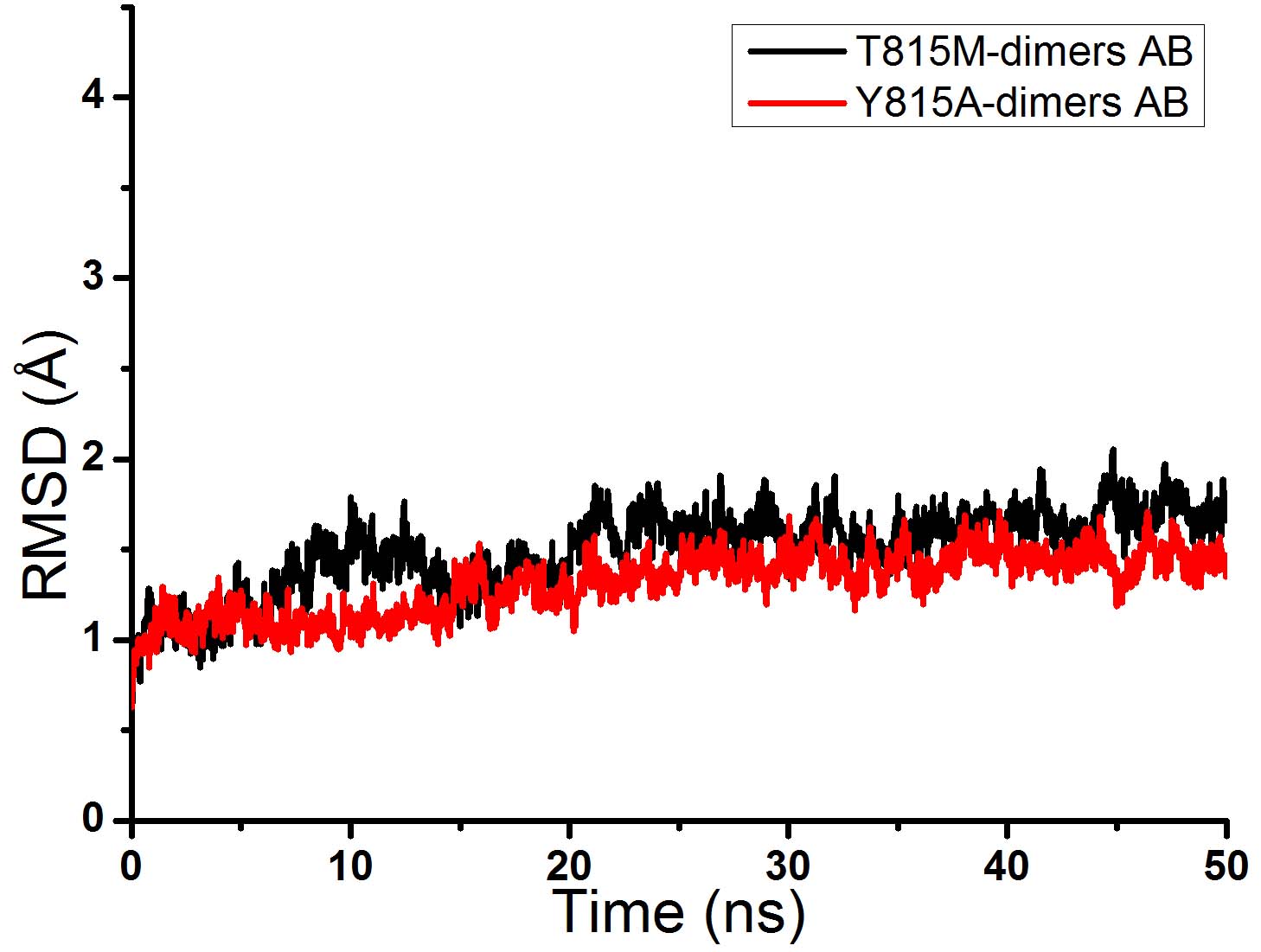


**Figure S3.** The RMSDs of backbone atoms of dimer A and B of T815M and Y805A mutants of mGlu_1_ versus simulated time.


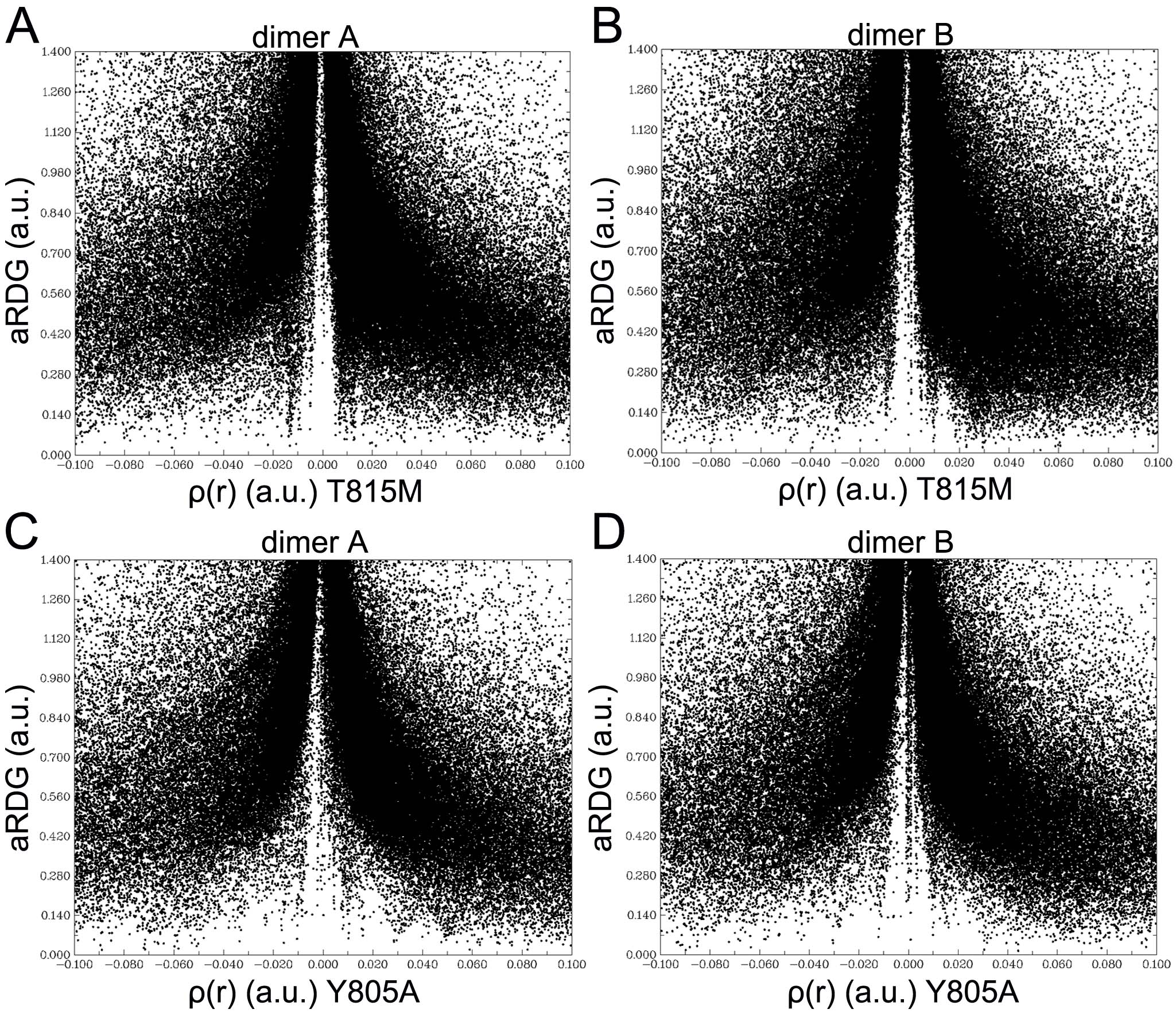


**Figure S4.** The averaging reduced density gradient (aRDG) versus averaging effective density between FITM and dimer A, B in mutated T815M and Y805A mutated mGlu_1_, respectively.
